# Inhibition of autophagy increased AGE/ROS-mediated apoptosis in mesangial cells

**DOI:** 10.1038/cddis.2016.322

**Published:** 2016-11-03

**Authors:** Li Xu, Qiuling Fan, Xu Wang, Xue Zhao, Lining Wang

**Affiliations:** 1 Department of Nephrology, First Hospital of China Medical University, Shenyang, China; 2 Department of Gastroenterology, First Hospital of China Medical University, Shenyang, China

## Abstract

The aim of our study was to investigate the role of autophagy, a homeostatic process involved in the lysosomal degradation of damaged cell organelles and proteins, in regulating the survival of mesangial cells treated with advanced glycation end products (AGEs). In the present study, AGEs induced mitochondrial depolarization and led to mitochondrial-dependent apoptosis in mesangial cells, as shown by the loss of the mitochondrial membrane potential; increased Bax processing; increased Caspase-9, Caspase-3 and PARP cleavage; and decreased Bcl-2 expression. Meanwhile, AGEs also triggered autophagy flux in mesangial cells, as confirmed by the presence of autophagic vesicles, the conversion of LC3II/LC3I and the increase/decrease in Beclin-1/p62 expression. Interestingly, this study reported apparent apoptosis and autophagy that were dependent on reactive oxygen species (ROS) production. Scavenging ROS with *N*-acetyl-l-cysteine could prevent the appearance of the autophagic features and reverse AGE-induced apoptosis. Moreover, AGE-triggered mitophagy, which was confirmed by the colocalization of autophagosomes and mitochondria and Parkin translocation to mitochondria, played a potential role in reducing ROS production in mesangial cells. Additionally, inhibition of autophagy significantly enhanced AGE-induced cell apoptosis. Taken together, our data suggest that ROS were the mediators of AGE-induced mesangial cell apoptosis and that autophagy was likely to be the mechanism that was triggered to repair the ROS-induced damage in the AGE-treated cells and thereby promote cell survival. This study provides new insights into the molecular mechanism of autophagy involved in AGE-induced apoptosis in mesangial cells.

The number of people with diabetes will increase from 382 million in 2013 to 592 million by 2035 worldwide, with 80% of cases occurring in low-income and middle-income countries of the International Diabetes Federation (IDF) Diabetes Atlas.^1^ Diabetic nephropathy (DN) is one of the most serious microvascular complications of diabetes and contributes to end-stage renal disease. However, the pathogenesis of DN is very complicated, and the underlying mechanisms remain incompletely resolved. Glomerular mesangial cells are known to play an important role in maintaining both the structure and functions of glomerular tufts. Studies have suggested that mesangial cell apoptosis is correlated with worsening albuminuria,^2^ and that the loss of mesangial cells through apoptosis contributes to diabetic glomerulosclerosis, which is involved in the pathogenesis and progression of DN.^3, 4^ Thus, a better understanding of mesangial cell apoptosis is required.

Advanced glycation end products (AGEs) are the end products of non-enzymatic glycosylation reactions between glucose and proteins, lipids and nucleic acids.^5^ During aging, renal failure, inflammation and particularly diabetes, the formation and deposition of AGEs are accelerated. Numerous data indicate that AGEs play an important role in the pathogenesis of DN.^6, 7, 8^ Patients with DN exhibit increased serum AGEs levels and decreased clearance.^9^ Immunohistochemical studies in patients with DN have shown that AGEs accumulate in the mesangium and glomerular capillary wall.^10^ In addition, both *in vivo* and *in vitro* studies showed that AGEs induce mesangial cell dysfunction and lead to apoptosis, which disturbs glomerular homeostasis and is involved in the pathogenesis of DN.^11, 12, 13^ However, the exact mechanisms of AGE-induced mesangial cell apoptosis are still unclear.

Autophagy is the primary metabolic process by which eukaryotic cells degrade and recover damaged macromolecules and organelles.^14^ During this process, substances in the cytoplasm are phagocytosed by autophagosomes, which are spherical structures with double layer membranes, and transported to the lysosomes for degradation. The degradation products can be re-used in the syntheses of macromolecules and in energetic metabolism.^14^ Autophagy is an important process that maintains cellular integrity and intracellular homeostasis during metabolic stress conditions. In fact, there is compelling evidence suggesting a close interplay between autophagy and apoptosis.^15, 16^ Although it has been shown that AGEs lead to mesangial cell apoptosis,^11, 12, 13^ it is not known whether autophagy is induced in AGE-caused mesangial cell apoptosis and, if so, how autophagy contributes to cell apoptosis. In this study, we investigated the molecular mechanism of mesangial cell apoptosis and the changes in autophagy flux in AGE-treated mesangial cells to elucidate the role of autophagy in determining the fate of AGE-treated mesangial cells.

## Results

### AGEs induced apoptosis in mesangial cells

**Figure 1:**
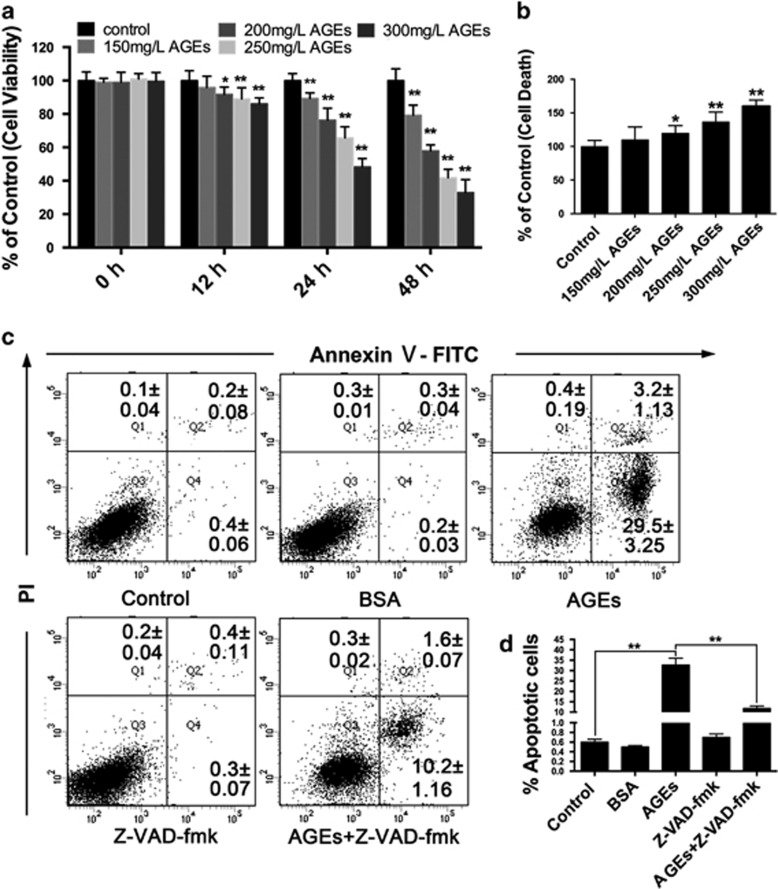
AGEs induced apoptosis in mesangial cells. (**a**) Cells were treated with various concentrations of AGEs (150–300 mg/l) for 0, 12, 24 or 48 h, and cell viability was estimated using the MTT assay. The data are presented as the mean±S.E.M. from at least three independent experiments. *P<0.05 *versus* Control, **P<0.01 *versus* Control. (**b**) Cells were treated with various concentrations of AGEs (150–300 mg/l) for 24 h. Cell death was estimated using a cell death detection ELISA^PLUS^ assay. The data are presented as the mean±S.E.M. from at least three independent experiments. *P<0.05 *versus* Control, **P<0.01 *versus* Control. (**c**) Cells were pre-treated with or without Z-VAD-fmk (25 *μ*M) and incubated with AGEs (250 mg/l) for 24 h. Cell apoptosis was detected using the annexin V-FITC/PI kit. Viable cells (annexin V^−^/PI^−^), early apoptotic cells (annexin V^+^/PI^−^), late apoptotic cells and necrotic cells (annexin V^+^/PI^+^) are located in the bottom left, bottom right and top right quadrants, respectively. The numbers in each quadrant represent the percentage of cells. The data are presented as the mean±S.E.M. from at least three independent experiments. (**d**) Percentages of apoptotic cells in each group. The data are presented as the mean±S.E.M. from at least three independent experiments. **P<0.01

We first treated cells with different concentrations of AGEs or bovine serum albumin (BSA) (150–300 mg/l) for different periods of time (0, 12, 24 and 48 h) and then evaluated cell viability with the 3-(4,5-dimethylthiazol-2-yl)-2,5-diphenyl-tetrazoliumbromide (MTT) assay to determine the effects of AGEs on mesangial cells. The results showed that AGEs decreased cell viability in a concentration-dependent and time-dependent manner, and the effects of AGEs were markedly significant beginning at 24 h (*P*<0.01) (Figure 1a). Treatments with various concentrations of the BSA control (150–300 mg/l) for different times (0, 12, 24 and 48 h) did not have an effect on cell viability (Supplementary Figure S1A). Additionally, to determine whether the inhibitory effect of AGEs on cell viability was related to cell apoptosis, a cell death detection ELISA^PLUS^ assay and an annexin V-FITC/PI apoptosis detection kit were used. The data from the cell death detection ELISA^PLUS^ assay showed that AGEs (200 mg/l) treatment for 24 h increased the DNA fragmentation ratio compared with the control group (*P*<0.05) and showed the most significant increase with 250 mg/l AGEs (*P*<0.01) (Figure 1b). Therefore, we chose 250 mg/l AGEs for all subsequent experiments. Treatments with various concentrations of the BSA control (150–300 mg/l) did not have an effect on cell death (Supplementary Figure S1B). The result of the annexin V-FITC/PI apoptosis detection assay showed that the percentage of apoptotic cells in the AGE-treated cells was remarkably increased compared with the control group (*P*<0.01). However, pre-treatment with a pan-caspase inhibitor, Z-VAD-fmk (*N*-benzyloxycarbonyl-Val-Asp (O-Me) fluoromethylketone; 25 *μ*M), prior to AGEs treatment significantly prevented the effect of AGEs on inducing cell apoptosis (*P*<0.01) (Figures 1c and d). These results suggested that AGEs induced the apoptotic death of mesangial cells.

### AGEs promoted ROS generation and triggered the mitochondrial apoptotic pathway

**Figure 2:**
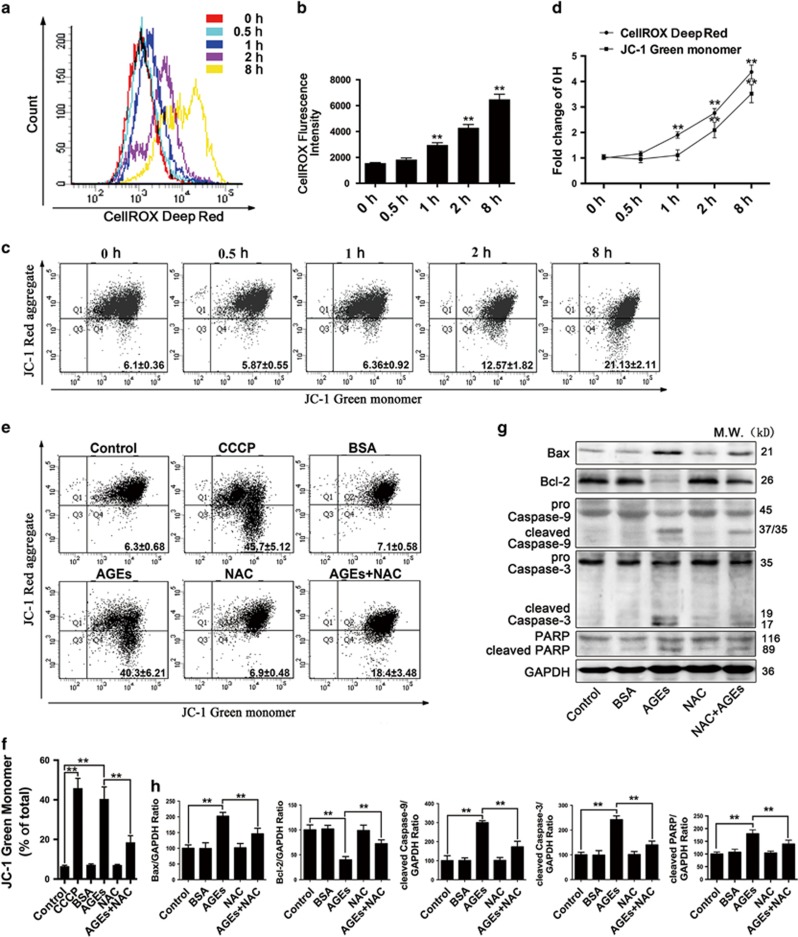
AGEs caused ROS-mediated mitochondrial depolarization and led to mitochondrial-dependent apoptosis in mesangial cells. (**a**–**d**) Cells were treated with AGEs (250 mg/l) for 0, 0.5, 1, 2 and 8 h. (**a**) The ROS levels were assessed by determining CellROX Deep Red fluorescence intensity via flow cytometry. (**b**) Quantitative analysis of fluorescence intensity. The data are presented as the mean±S.E.M. from at least three independent experiments. **P<0.01 *versus* 0 h. (**c**) The level of MMP was determined by flow cytometric analysis of the JC-1 dye. The numbers in each quadrant represent the green (monomer) fluorescence ratio. The data are presented as the mean±S.E.M. from at least three independent experiments. (**d**) Time-kinetics analysis of (**a**) and (**b**). The data are presented as the mean±S.E.M. from at least three independent experiments. **P<0.01 *versus* 0 h. (**e**–**h**) Cells were pre-treated with or without NAC (5 mM) and then incubated with AGEs (250 mg/l) for 24 h. (**e**) The level of MMP was determined by flow cytometric analysis of the JC-1 dye. CCCP, a mitochondrial membrane potential disrupter, was used as a positive control. The numbers in each quadrant represent the green (monomer) fluorescence ratio. The data are presented as the mean±S.E.M. from at least three independent experiments. (**f**) Quantification of JC-1 green fluorescence. The data are presented as the mean±S.E.M. from at least three independent experiments. **P<0.01. (**g**) Western blot analysis of Bax, Bcl-2, Caspase-9, Caspase-3 and PARP expression in each group. (**h**) Quantitative analysis of Bax, Bcl-2, cleaved Caspase-9, cleaved Caspase-3 and cleaved PARP protein expression. The data are presented as the mean±S.E.M. from at least three independent experiments. **P<0.01

Intracellular reactive oxygen species (ROS) play a critical role in different types of cell survival.^17, 18, 19^ We assessed the ROS levels by determining the CellROX Deep Red fluorescence intensity via flow cytometry to investigate the effect of AGEs on ROS generation. A time-course experiment showed that ROS generation occurred as early as 1 h and remained stable in AGE-treated cells (*P*<0.01) (Figures 2a and b). Because a rise in intracellular ROS levels can damage various cell components, including mitochondria,^18, 19^ we next explored ROS-induced mitochondrial dysfunction. We used flow cytometric analysis of cells loaded with the potentiometric dye JC-1 to assess changes in mitochondrial membrane potential (MMP). We found that AGE-treated cells exhibited increased accumulation of the JC-1 monomers beginning at 2 h (*P*<0.01) (Figure 2c). The time-kinetics analysis revealed that the increase in ROS generation preceded the increase in the accumulation of JC-1 monomers in AGE-treated cells (Figure 2d). Additionally, mesangial cells were exposed to the uncoupler CCCP (10 *μ*M) as a positive control, resulting in a significant accumulation of JC-1 monomers. Pre-treatment with *N*-acetyl-l-cysteine (NAC) (5 mM), a ROS scavenger, significantly stabilized the MMP, resulting in a reduced accumulation of JC-1 monomers (*P*<0.01) (Figures 2e and f).

The mitochondrial pathway of apoptosis begins with the depolarization of MMP, which is mainly regulated by Bcl-2 family members. Subsequently, the reduction of the MMP activates Caspase-9, a mitochondrial-dependent apoptotic initiator, which in turn activates Caspase-3, leading to apoptosis.^19, 20^ Therefore, we examined the expression of apoptotic-related molecules by western blot analysis. The results showed that AGEs treatment for 24 h significantly increased the expression of the proapoptotic protein-Bax, cleaved Caspase-9, cleaved Caspase-3 and cleaved PARP but decreased the expression of antiapoptotic proteins, such as Bcl-2, compared with the control group (*P*<0.01). Pre-treatment with 5 mM NAC reversed the effect of AGEs on the expression of apoptosis-related molecules (*P*<0.01) (Figures 2g and h). Collectively, these data indicated that AGEs induced ROS generation, mediated the loss of the MMP and contributed to mitochondrial apoptosis in mesangial cells.

### AGEs induced autophagy flux in mesangial cells

**Figure 3:**
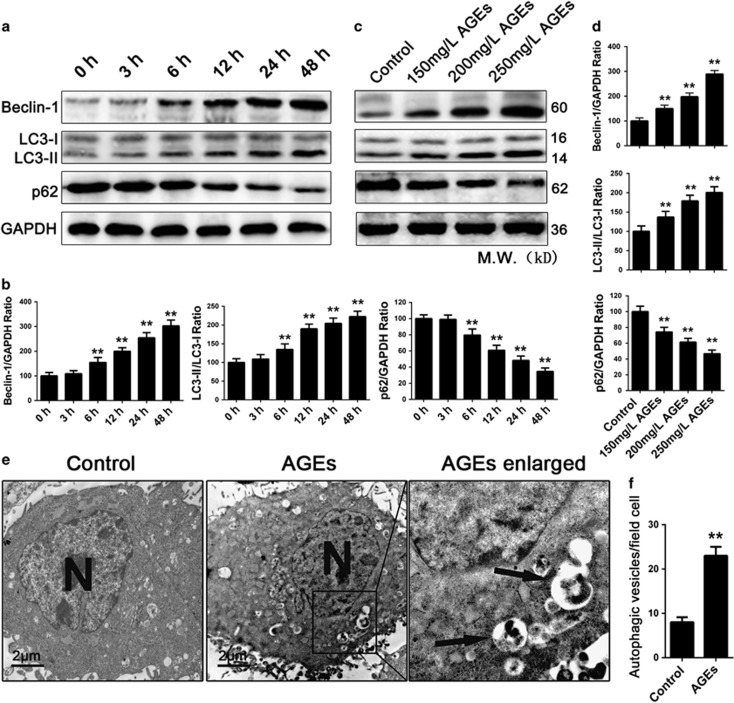
AGEs affected the expression of autopaghy-related proteins and promoted the formation of autophagic vesicles in mesangial cells. (**a**) Western blot analysis of Beclin-1, LC3II/LC3I and p62 protein levels in mesangial cells treated with 250 mg/l AGEs for 0, 3, 6, 12, 24, 48 h. (**c**) Western blot analysis of Beclin-1, LC3II/LC3I and p62 protein levels in mesangial cells treated with various concentrations of AGEs (150–250 mg/l) for 24 h. (**b** and **d**) Quantitative analysis of Beclin-1, LC3II/LC3I and p62 protein expression. The data are presented as the mean±S.E.M. from at least three independent experiments. **P<0.01 *versus* 0 h or Control. (**e**) Transmission electron microscopy showed autophagic vesicles (bold arrows) in cells that had been treated with 250 mg/l AGEs for 24 h. Bar=2 *μ*m. (**f**) Quantification of the autophagic vesicles in 10 randomly selected cells. The data are presented as the mean±S.E.M. **P<0.01 *versus* Control

Next, we examined whether AGEs induce autophagy in mesangial cells. LC3II and Beclin-1 are well-known biomarkers of the formation of autophagosomes. In addition, p62 is a biomarker of the degradation of autolysosomes.^21^ We treated cells with different concentrations of AGEs for different times and analyzed the Beclin-1, LCII/LC3I and p62 levels by western blotting to determine the effect of AGEs on autophagy in mesangial cells. Compared with the control group, AGEs increased the Beclin-1 levels and LC3II/LC3I conversion and decreased the p62 levels in a time-dependent manner (*P*<0.01) (Figures 3a and b). With increased concentrations of AGEs, the expression of Beclin-1 and LC3II/LC3I increased and the expression of p62 decreased accordingly compared with the control group (*P*<0.01), which suggested that the effect was also concentration-dependent (Figures 3c and d). We used a transmission electron microscope (TEM) to count the number of autophagic vesicles to assess whether the change in the expression levels of autophagy-related proteins was also associated with an increase in the formation of autophagic vesicles. After a 24 h treatment with AGEs (250 mg/l), we observed a significant increase in the number of autophagosomes/autophagolysosomes in mesangial cells compared with the control group (*P*<0.01) (Figures 3e and f).

**Figure 4:**
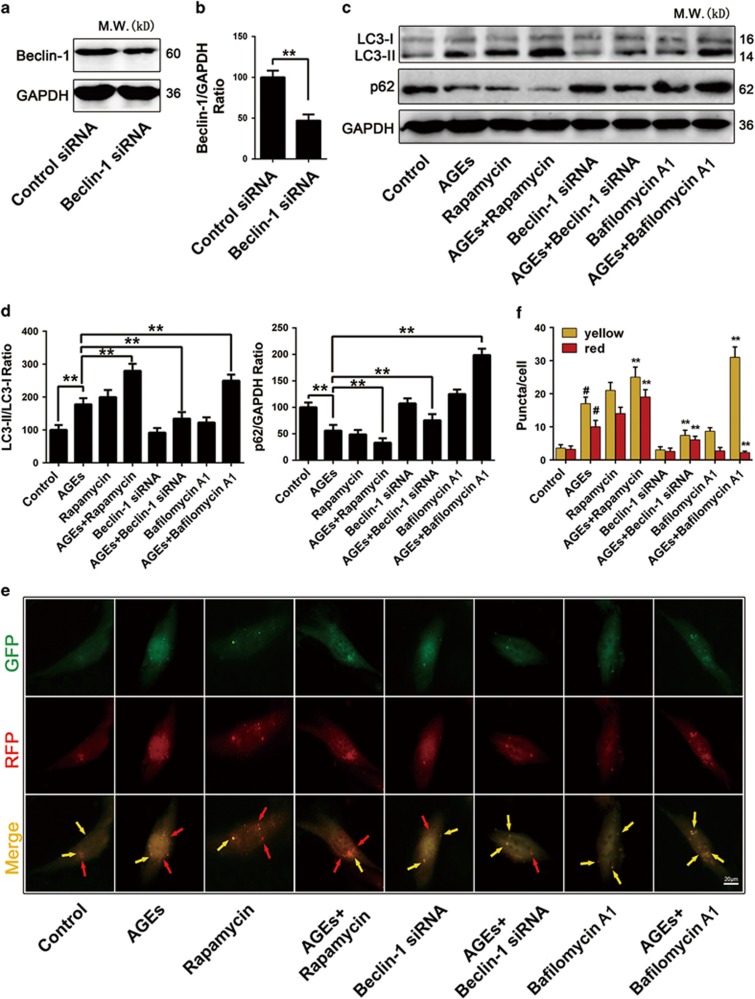
Autophagy flux measurements in AGE-treated mesangial cells. (**a**) Western blot analysis of Beclin-1 protein levels in mesangial cells that were transiently transfected with control siRNA or Beclin-1 siRNA for 24 h. (**b**) Quantitative analysis of Beclin-1 protein expression. The data are presented as the mean±S.E.M. from at least three independent experiments. **P<0.01. (**c**) Western blot analysis of LC3II/LC3I and p62 protein levels in mesangial cells treated with or without Rapamycin (100 nM), Bafilomycin A1 (10 nM) or Beclin-1 siRNA and incubated with or without AGEs (250 mg/l) for 24 h. (**d**) Quantitative analysis of LC3II/LC3I and p62 protein expression. The data are presented as the mean±S.E.M. from at least three independent experiments. **P<0.01. (**e**) Mesangial cells were transiently transfected with the GFP-RFP-LC3 plasmid for 24 h and then further treated with or without Rapamycin (100 nM), Bafilomycin A1 (10 nM) or Beclin-1 siRNA and incubated with AGEs (250 mg/l) for 24 h. Arrowheads: yellow puncta (RFP^+^/GFP^+^-LC3 puncta), red puncta (RFP^+^/GFP^−^-LC3 puncta). Bar=2 *μ*m. (**f**) The yellow and red puncta were quantified (>20 cells were counted in each experiment, and at least three independent experiments were performed). The data are presented as the mean±S.E.M. ^#^*P*<0.01 *versus* control, **P<0.01 *versus* AGE-treated cells

Currently, it is believed that analyzing the number of autophagic vesicles alone is not an adequate method of measuring autophagic degradation activity (flux) because a growing number of autophagy-related structures can indicate increased generation and decreased clearance.^21^ Thus, we validated the effects of AGEs on LC3II/LC3I conversion and p62 protein expression at 24 h in the presence/absence of the pharmacological autophagy activator Rapamycin (100 nM), an autophagic-lysosomal degradation inhibitor, Bafilomycin A1 (10 nM), and a genetic inhibitor of autophagy, Beclin-1 siRNA. The results showed that Beclin-1 siRNA could significantly decrease Beclin-1 expression (*P*<0.01) (Figures 4a and b). Compared with the AGE-treated group, pre-treatment with Rapamycin prior to AGEs treatment further increased LC3II/LC3I conversion and decreased p62 expression (*P*<0.01). Moreover, siRNA-mediated knockdown of Beclin-1 decreased LC3II/LC3I conversion and increased p62 expression (*P*<0.01). However, pre-treatment with Bafilomycin A1 prior to AGEs treatment increased the expression of both LC3II/LC3I and p62 (*P*<0.01) (Figures 4c and d), suggesting decreased autophagosome clearance. We utilized an expression vector encoding LC3 fused to red fluorescent protein (RFP) and green fluorescent protein (GFP) in tandem (mRFP-GFP-LC3) to further assess autohpagy flux. Compared with the control group, a 24 h treatment with AGEs significantly increased both the yellow and red puncta (*P*<0.01). Compared with the AGE-treated group, pre-treatment with Rapamycin further increased the yellow and red puncta. However, Beclin-1 siRNA decreased both the yellow and red puncta. Furthermore, Bafilomycin A1 significantly increased the yellow puncta but decreased red puncta (all *P*<0.01) (Figures 4e and f). Taken together, these findings firmly showed that AGEs induced autophagy flux in mesangial cells.

### The ROS-mediated ERK signaling pathway was involved in AGE-induced autophagy in mesangial cells

**Figure 5:**
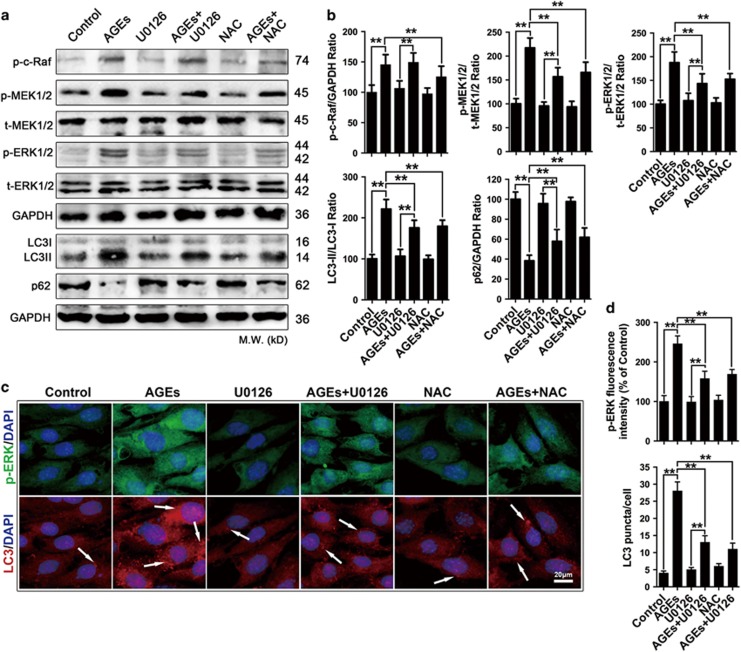
AGE-induced mesangial cell autophagy was mediated by the ROS/ERK signaling pathway. (**a**–**d**) Cells were pre-treated with or without U0126 (30 *μ*M) or NAC (5 mM) and incubated with AGEs (250 mg/l) for 24 h. (**a**) Western blot analysis of p-c-Raf, p-MEK1/2, t-MEK1/2, p-ERK1/2 and t-ERK1/2 protein expression levels and autophagy-related protein (LC3II/I and p62) levels in mesangial cells. (**b**) Quantitative analysis of p-c-Raf, p-MEK1/2, p-ERK1/2, LC3II/I and p62 protein expression levels. The data are presented as the mean±S.E.M. from at least three independent experiments. **P<0.01. (**c**) LC3/p-ERK expression was assessed by fluorescent microscopy. Arrowhead: LC3 puncta accumulation. Bar=2 *μ*m. (**d**) Quantitative analysis of p-ERK fluorescence intensity and LC3 puncta. The data are presented as the mean±S.E.M. from at least three independent experiments. **P<0.01

Previous studies have indicated that the ROS-mediated ERK pathway was responsible for the induction of autophagy.^22, 23^ We next determined the effect of the ROS/ERK signaling pathway on autophagy levels in AGE-treated cells. Firstly, we investigated the expression of proteins in the ERK signaling pathway and autophagy process in the absence or presence of the MEK inhibitor U0126 and NAC using western blotting. The results showed that treatment with AGEs for 24 h increased the expression of phospho-c-Raf (p-c-Raf), p-MEK1/2 and p-ERK1/2; LC3II/LC3I conversion was concomitantly increased, and p62 expression was decreased (*P*<0.01) (Figures 5a and b). Furthermore, in cells that were pre-treated with U0126 (30 *μ*M) before being exposed to AGEs, U0126 reduced the expression of p-MEK1/2 and p-ERK1/2, decreased LC3II/LC3I conversion, and increased p62 expression (*P*<0.01) but did not affect p-c-Raf expression (Figures 5a and b). In cells that were incubated with NAC prior to treatment with AGEs, NAC reversed the effect of AGEs on ERK signaling pathway and autophagy process (*P*<0.01) (Figures 5a and b). Next, we examined ERK expression and endogenous LC3 puncta in mesangial cells by immunofluorescence analysis. Upon AGEs treatment for 24 h, the p-ERK level increased and the number of endogenous LC3 puncta increased concomitantly compared with controls. Pre-treatment with U0126 and NAC prior to AGEs treatment markedly decreased the p-ERK levels and the number of endogenous LC3 puncta (all *P*<0.01) (Figures 5c and d). Taken together, all of these findings proved that the ROS-mediated ERK signaling pathway had a role mesangial cell autophagy induced by AGEs.

### Mitophagy had a potential role in reducing ROS production in AGE-treated mesangial cells

**Figure 6:**
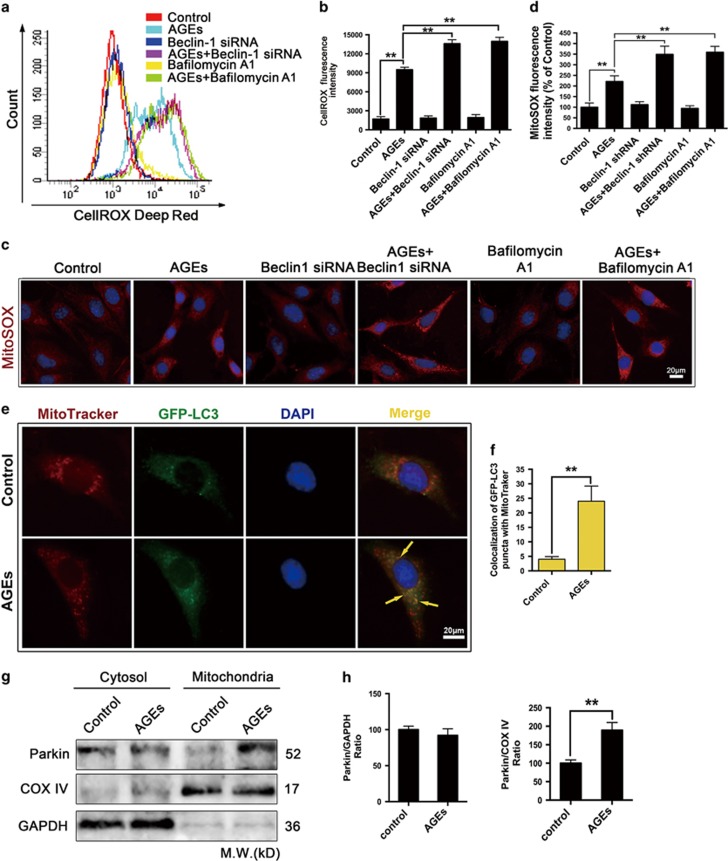
AGEs triggered autophagic clearance of ROS. (**a**–**d**) Cells were pre-treated with or without Beclin-1 siRNA and Bafilomycin A1 (10 nM) and then incubated with AGEs (250 mg/l) for 24 h. (**a**) The ROS levels were assessed by determining CellROX Deep Red fluorescence intensity via flow cytometry. (**b**) Quantitative analysis of the fluorescence intensity. The data are presented as the mean±S.E.M. from at least three independent experiments. **P<0.01. (**c**) Mitochondrial superoxide accumulation was determined by measuring the MitoSOX Red fluorescent intensity. Bar=2 *μ*m. (**d**) Quantitative analysis of the mean MitoSOX Red fluorescence (>20 cells were imaged in each experiment, and at least three independent experiments were performed). The data are presented as the mean±S.E.M. **P<0.01. (**e**–**h**) Cells were treated with AGEs (250 mg/l) for 24 H. (**e**) Immunofluorescence analysis was used to determine the number of GFP-LC3-positive autophagosomes that colocalized with MitoTracker-labeled mitochondria. Arrowhead: yellow arrows indicate colocalization. Bar=2 *μ*m. (**f**) Quantitative analysis of GFP-LC3/MitoTracker colocalization as an index of mitophagy (>20 cells were imaged in each experiment, and at least three independent experiments were performed). The data are presented as the mean±S.E.M. **P<0.01. (**g**) Cytosolic and mitochondrial fractions were isolated from the cells, and Parkin expression was analyzed by western blotting. GAPDH and COX IV were used as loading controls for the cytosolic and mitochondrial fractions, respectively. (**h**) Quantitative analysis of Parkin protein expression. The data are presented as the mean±S.E.M. from at least three independent experiments. **P<0.01

We examined the effect of inhibiting autophagy on the potential AGE-mediated ROS generation to determine the biological significance of autophagy in mesangial cells in response to AGEs. Interestingly, although ROS were responsible for AGE-triggered autophagy (Figure 5), the present data showed that autophagy inhibition by Beclin-1 siRNA and bafilomycin A1 induced a further increase in ROS levels compared to AGE-treated cells (*P*<0.01) (Figures 6a and b), suggesting that the increase in ROS levels might be generated from different sources in the AGE-treated cells. It is well established that ROS-damaged mitochondria lead to the production of more ROS, which in turn aggravates mitochondria damage and other intracellular abnormalities.^24, 25^ The cells were loaded with MitoSox Red, an indicator of mitochondrial superoxide levels, to examine whether AGEs induced the generation of mitochondrial ROS. The MitoSox data were in accord with the CellROX data (Figures 6c and d). All of the data showed that AGEs induced intracellular ROS and mitochondrial ROS generation, accompanied by their autophagic clearance.

The selective engulfment of mitochondria and removal of mitochondria is referred to as mitophagy. Mitophagy can remove damaged mitochondria and reduce ROS production.^25, 26^ The cells were transiently transfected with GFP-LC3, and colocalization of GFP-LC3 with MitoTracker Red was analyzed as an index of mitophagy to investigate whether AGEs cause mitophagy in mesangial cells. Fluorescence microscopy revealed extensive colocalization between GFP-LC3 puncta (green) and MitoTracker-labeled mitochondria (red) in AGE-treated cells, but little colocalization was observed in control cells (*P*<0.01) (Figures 6e and f). Moreover, we utilized western blotting to analyze the expression of Parkin, which is recruited to dysfunctional mitochondria with low membrane potential and initiates mitophagy.^27^ The data showed that Parkin was not present in the mitochondria of control cells but was translocated into the mitochondria of AGE-treated cells (*P*<0.01) (Figures 6g and h). Taken together, these results suggest that mitophagy might play an essential role in the autophagic clearance of ROS in AGE-treated cells.

### Autophagy as a protective response against AGE-induced apoptosis in mesangial cells

**Figure 7:**
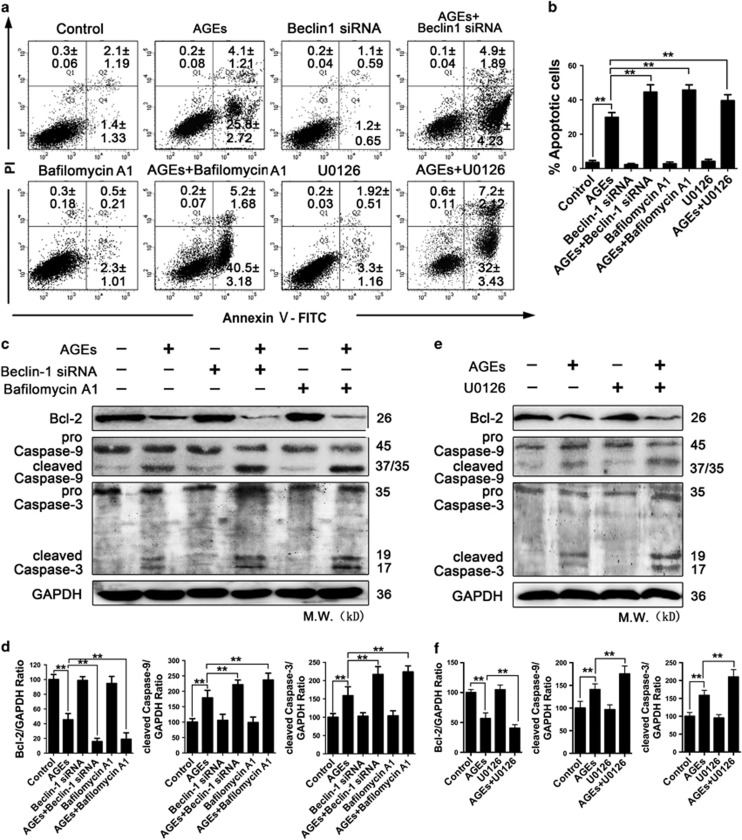
Inhibition of autophagy aggravated AGE-induced mesangial cell apoptosis. (**a**–**d**) Cells were pre-treated with or without Beclin-1 siRNA and Bafilomycin A1 (10 nM) and then incubated with AGEs (250 mg/l) for 24 h. (**a**) Cell apoptosis was detected using the annexin V-FITC/PI kit. The numbers in each quadrant represent the percentage of cells. The data are presented as the mean±S.E.M. from at least three independent experiments. (**b**) Percentages of apoptotic cells in each group. The data are presented as the mean±S.E.M. from at least three independent experiments. **P<0.01. (**c**) Western blot analysis of Bcl-2, Caspase-9 and Caspase-3 expression in each group. (**d**) Quantitative analysis of Bcl-2, cleaved Caspase-9 and cleaved Caspase-3 protein expression. The data are presented as the mean±S.E.M. from at least three independent experiments. **P<0.01. (**e** and **f**) Cells were pre-treated with or without U0126 (30 *μ*M) and incubated with AGEs (250 mg/l) for 24 h. (**e**) Western blot analysis of Bcl-2, Caspase-9 and Caspase-3 expression in each group. (**f**) Quantitative analysis of Bcl-2, cleaved Caspase-9 and cleaved Caspase-3 protein expression. The data are presented as the mean±S.E.M. from at least three independent experiments. **P<0.01

Autophagy represents a double-edged sword in cell fate and includes pro-survival and pro-death activities.^15, 16, 28, 29, 30^ We exposed mesangial cells to a combination of AGEs and autophagy inhibitors (Beclin-1 siRNA and Bafilomycin A1) and examined apoptotic cell death to determine the role of autophagy in AGE-mediated apoptotic cell death. The results showed that Beclin-1 siRNA and Bafilomycin A1 increased the percentage of apoptosis cells induced with AGEs (*P*<0.01) (Figures 7a and b). Furthermore, we determined the expression of apoptosis-related molecules by western blotting. Compared with AGE-treated cells, Beclin-1 siRNA and Bafilomycin A1 further decreased Bcl-2 levels, increased cleaved caspase-3 and cleaved caspase-9 levels (*P*<0.01) (Figures 7c and d). Additionally, we further investigated the effect of U0126 on cell apoptosis in AGE-treated cells. The results were consistent with the results of the autophagy inhibitors (Figures 7a, b, e and f). These results suggested that AGE-induced cell apoptosis was aggravated by autophagy inhibition, and that autophagy has a protective effect on AGE-induced apoptosis in mesangial cells.

## Discussion

**Figure 8:**
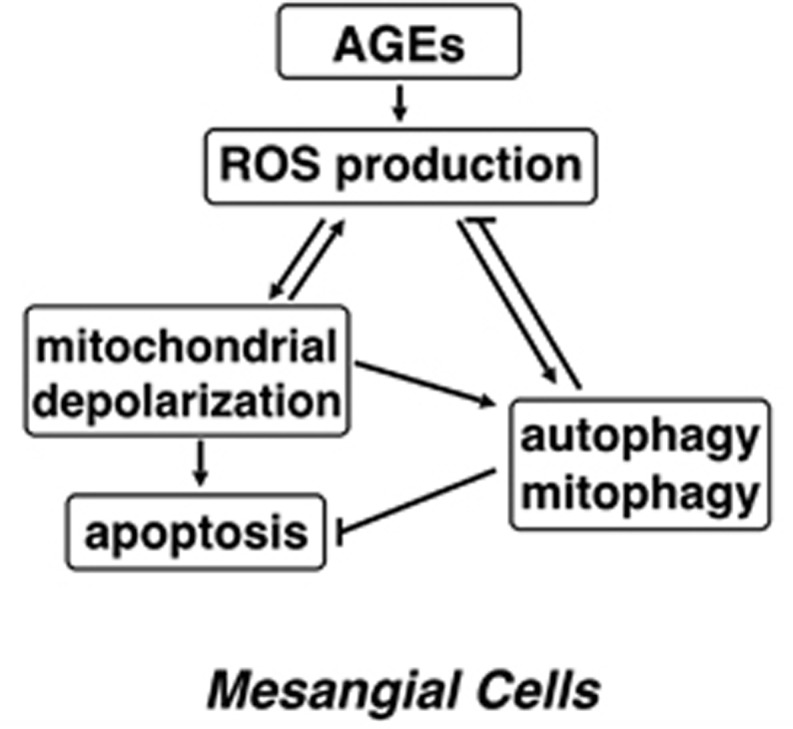
Proposed model depicting the mechanism of action of AGEs in mesangial cells

Mesangial cells occupy a central anatomical position in the glomerulus. In fact, they actually provide structural support for capillary loops and modulate glomerular filtration through their smooth muscle activity.^31^ Therefore, AGE-induced mesangial cells loss and the resulting altered mesangial cell contractility may contribute in part to glomerular hyperfiltration.^11^ Here, we reported that ROS have a key role in mitochondrial-mediated mesangial cell apoptosis induced by AGEs. Interestingly, ROS production could also trigger autophagy, which appears to be an important protective mechanism against AGE-mediated apoptosis. Based on these findings, we propose a model depicting the possible mechanism of action of AGEs in mesangial cells (Figure 8).

Notably, a receptor for AGE (RAGE) appears to be responsible for renal cell death, such as podocytes^13^ and tubular cells,^32^ because apoptosis was fully inhibited by blocking RAGE with the anti-RAGE antibody. In contrast, RAGE inhibition did not block mesangial cell apoptosis.^11, 13^ Therefore, the results suggest that mesangial cells appear to have non-RAGE-related mechanisms to induce death in response to AGEs. ROS are highly reactive oxygen free radicals or non-radical molecules that have essential roles in deciding cell fate.^17, 18^ AGEs increase the levels of ROS through activation of NADPH oxidase^33, 34^ and mitochondrial pathway^35^ in both a receptor-dependent manner (i.e., through the advanced glycation end product receptor, RAGE) and a receptor-independent manner.^8^ In mesangial cells, AGEs markedly boosted ROS production, upregulated proinflammatory cytokine production, such as interleukin-6, monocyte chemoattractant protein-1, intercellular adhesion molecule-1^36, 37^ and transforming growth factor-*β*1, and induced fibronectin overproduction,^38, 39^ which initiates and participates in the progression of diabetic renal fibrosis. On the other hand, evidence has shown that AGE incubation decreased superoxide dismutase activity and led to mesangial cell apoptosis.^12^ In addition, a ROS scavenger, NAC, dramatically prevented AGE-induced mesangial cell apoptosis.^11^ It seems that ROS were responsible for AGE-induced mesangial cell apoptosis. However, the exact mechanisms of ROS in mesangial cells triggered by AGEs are not well addressed. The findings of the present study further extend these previous studies and demonstrate first that AGE-induced ROS generation is a relatively early event, suggesting that ROS could be the mediator that leads to the loss of MMP and the subsequent mitochondria-dependent apoptosis in mesangial cells, which provides a theoretical basis for the molecular pathogenesis of AGE-induced mesangial cell apoptosis.

There is abundance evidence indicating that AGEs increase autophagy in different cell types.^40, 41^ However, the effect of AGEs on autophagy in mesangial cells is unknown. Because it is difficult to monitor autophagy,^21^ we have conducted a series of experiments to examine autophagy-related markers in AGE-treated mesangial cells, including LC3 conversion, autophagic vesicle formation and the increase/decrease in expression of several autophagy-related proteins (Beclin-1/p62). Furthermore, we examined the autophagy process by employing the tandem fluorescent construct-GFP-RFP-LC3. All of these results confirmed the ability of AGEs to induce the autophagic response in mesangial cells.

It is well established that enhanced ROS production induces autophagy,^22, 42, 43^ which was confirmed in the present study. We found that treatment with the ROS scavenger NAC reversed LC3 conversion and puncta and decreased p62 expression in AGE-treated cells, suggesting that AGE-induced autophagy was related to ROS accumulation in mesangial cells. Many lines of evidence suggest that the activation of ERK signaling pathway is responsible for ROS-triggered autophagy in various cell types.^22, 23^ It has been reported that ROS regulate the Ras/Raf/ERK signaling pathway to modulate downstream AP-1 binding gene expression.^44^ Additionally, recent studies have shown that the Raf/MEK/ERK signaling pathway participates in the regulation of autophagy by regulating LC3 and p62 expression.^45^ Although it has been shown that AGEs activate the ERK signaling pathway in mesangial cells, autophagy has not been assessed.^46^ In the present study, the AGE-mediated increase in expression of autophagy-related proteins was associated with modulation of ERK signaling pathway activity. The MEK inhibitor U0126 reversed the effect of AGEs on the expression of p-MEK and p-ERK and autophagy of mesangial cells, but did not affect the expression of c-Raf. Interestingly, a ROS scavenger NAC inhibited the phosphorylation of c-Raf and MEK and prevented the activation of the ERK signaling pathway. The results suggest that the ERK signaling pathway, which triggers autophagy, functions as a downstream signal of ROS in AGE-treated mesangial cells. These results provide new insight to allow us to understand the mechanism by which AGE-mediated signaling leads to the activation of autophagy.

To date, several studies have shown that autophagy is a homeostatic process that serves as a cytoprotective mechanism, allowing cells to survive and escape apoptosis induced by diverse stress conditions.^28, 29, 41^ However, little is known about the function of autophagy in mesangial cells under stress conditions. Although increased ROS production triggered autophagy in AGE-treated cells, we observed increased intracellular ROS and mitochondrial ROS generation in mesanigal cells, accompanied by increased autophagic clearance. Furthermore, the results presented here also showed that AGE-induced autophagy involved mitophagy. This finding is consistent with the proposal suggesting that the ROS generated from damaged mitochondria lead to the collapse of the MMP and a transient increase in ROS generation via the electron transfer chain, which constitutes a positive feedback circle,^24^ eventually leading to autophagic clearance of the impaired mitochondria,^42^ and a reduction in oxidative stress.^26^ In addition to mitophagy, general autophagy confers a cytoprotective role. General autophagy facilitates the maintenance of ATP and furnishes the basic building blocks for the synthesis of proteins that inhibit apoptosis. Moreover, general autophagy also eliminates qtoxic protein aggregates, inhibits proteasome function, affects the progression of the cell cycle and liberates antiapoptotic proteins, such as BCL-2 and the caspase-8 inhibitor FLIP from their inhibitory interactions with autophagic effectors.^47^ In the present study, we found that blocking autophagy with pharmacological and genetic inhibitors led to a remarkable increase in apoptosis in response to AGEs. Therefore, it is apparently that both AGE-triggered mitophagy and general autophagy may mediate cytoprotective effects on mesangial cells. However, the possible mechanisms of autophagy/mitophagy in the observed effect require further exploration.

Taken together, our studies provide experimental evidence that ROS have a key role in mediating AGE-induced apoptosis and autophagy in mesangial cells. Here, we identify an important protective role of autophagy such that ROS generation and cell apoptosis are markedly enhanced if the autophagic response is inhibited. These results suggest that treatments targeting autophagy in mesangial cells might be an alternative strategy for the therapy of DN.

## Materials and Methods

### Cell culture and materials

The rat mesangial cell line HBZY-1 was purchased from the China Center for Type Culture Collection (Wuhan, China), and the mesangial cells were cultured and cloned as previously described.^48^

BSA, MTT, Z-VAD-fmk, Rapamycin, Bafilomycin A1 and U0126 were obtained from Sigma (St. Louis, MO, USA). The mRFP-GFP-LC3 plasmid was purchased from Genechem (Shanghai, China). The GFP-LC3 plasmid was kindly provided by Addgene (Cambridge, MA, USA). Primary antibodies for Caspase-3, PARP, LC3, Beclin-1, p62, p-c-Raf, total ERK1/2 (t-ERK1/2), p-ERK1/2, p-MEK1/2, t-MEK, Parkin and COX IV were purchased from Cell Signaling Technology (Danvers, MA, USA), and antibodies for Bax, Bcl-2, Caspase-9 and GAPDH were purchased from Abcam (Cambridge, MA, USA).

### Preparation of AGE protein

BSA was added into 10 mM phosphate-buffered saline (PBS) (pH 7.4, concentration of 5 g/l) and incubated with 50 mM d-glucose in 5% CO_2_ air at 37 °C for 12 weeks. Unincorporated glucose was removed by dialysis overnight against PBS. AGE-BSA specific fluorescence determinations were performed by measuring emission at 440 nm on excitation at 370 nm using a fluorescence spectrophotometer (Hitachi, Tokyo, Japan). The fluorescence intensity of AGE-BSA was 50 times higher than BSA. AGE-BSA content was estimated by fluorescence intensity at a protein concentration of 1 mg/ml. AGE-BSA was stored at −70 °C until use.

### Cell viability analysis

Mesangial cells were seeded onto 96-well plates at a density of 3 × 10^3^ cells/well for 24 h, and the culture medium was replaced. After treatment, culture media were changed for serum-free culture media. MTT dissolved in PBS was added to each well and then incubated for 4 h. Then, the culture medium was removed, and dimethyl sulfoxide (DMSO) (Sigma) was added into each well. The cells were placed in an incubator and vortexed at a low speed for 10 min to fully dissolve the crystals. The optical densities were measured at 490 nm spectral wavelength using a microplate reader (Spectra Thermo, Tecan Group Ltd., Männedorf, Switzerland).

### Cell apoptosis analysis

A cell death detection apoptosis ELISA^PLUS^ assay (Roche, Basel, Switzerland) was performed to determine apoptosis by quantification of histone-complexed DNA fragments according to the instructions of manufacturer, and absorbance was determined at 405 nm wavelength using a microplate reader.

The mesangial cells apoptotic ratio was determined using the fluorescent dye annexin V-FITC/PI apoptosis detection kit according to the manufacturer's protocols (KeyGEN, Nanjing, China). Cells were harvested and washed in cold PBS twice, re-suspended in 500 *μ*l of binding buffer, and incubated with 5 *μ*l of annexin V-FITC and 5 *μ*l of PI solution for 15 min at room temperature in the dark, and then immediately analyzed by flow cytometry using a FACS flow cytometer equipped with Modfit LT 3.0 (BD Biosciences, San Jose, CA, USA). Approximately 1 × 10^4^ cells were analyzed in each of the samples.

### ROS measurement

ROS generation was determined using a CellROX Deep Red Flow Cytometry Assay Kit (Life Technologies, Carlsbad, CA, USA). The fluorescence intensity of CellROX Deep Red reflects the ROS levels. Briefly, cells were seeded on a six-well plate. After treatment, the cells were harvested with trypsin-EDTA (Sigma). The CellROX Deep Red reagent was added to the samples at a final concentration of 5 *μ*M and incubated for 30 min at 37 °C in the dark. The medium was then removed, and the cells were washed three times with PBS. Cells were then subjected to flow cytometry using a FACS flow cytometer equipped with Modfit LT 3.0 (BD Biosciences) (excitation/emission wavelengths: 640/665 nm). Approximately 1 × 10^4^ cells were analyzed in each of the samples.

Mitochondrial superoxide production was monitored using MitoSOX Red staining (Invitrogen, Carlsbad, CA, USA). Before use, MitoSOX Red was dissolved in DMSO to yield a 5 mM stock and subsequently diluted to a working concentration of 5 *μ*M in warm PBS. At 24  h after treatment, cells were treated with MitoSOX red (5 *μ*M) for 10 min at 37 °C in the dark. The cells were washed and incubated with DAPI (nuclear stain) for 5 min. Subsequently, the cells were visualized with a fluorescence microscope (Olympus, Tokyo, Japan).

### MMP measurement

MMP was evaluated using cationic dye JC-1 (Beyotime, Shanghai, China). In normal cells, JC-1 aggregates in the mitochondria as red fluorescent aggregates. In apoptotic cells, JC-1 accrues in the cytosol as green fluorescent monomers. The cells were seeded on a six-well plate. After treatment, cells were harvested and incubated with 100 *μ*l of 5,5′,6,6′-tetrachloro-1,1′,3,3′-tetraethyl-imidacarbocyanine iodide (JC-1) at a final concentration of 10 *μ*g/ml for 30 min at 37 °C. After staining, the cells were washed twice with cold PBS and subjected to flow cytometry analyses using a FACS flow cytometer equipped with Modfit LT 3.0 (BD Biosciences). Approximately 1 × 10^4^ cells were analyzed in each of the samples.

### Western blot analysis

After treatment, the cells were extracted with lysis buffer containing protease inhibitors (150 mM NaCl, 1% NP-40, 0.1% SDS, 2 *μ*g/ml aprotinin, 1 mM phenylmethanesulfonyl fluoride) for 30 min at 4 °C. The supernatants were centrifuged at 12 000 × * g* for 15 min at 4 °C. The supernatant containing total protein was harvested. Aliquots containing 20 *μ*g of proteins were separated by a 12% SDS-PAGE and transferred to polyvinylidene fluoride membranes. The membranes were soaked in blocking buffer (5% skimmed milk) for 2 h. Subsequently, proteins were detected using different primary antibodies overnight at 4 °C, then visualized using anti-goat or anti-rabbit IgG conjugated with horseradish peroxidase (Abcam) for 2 h at room temperature. The EC3 Imaging System (UVP, LLC, CA, USA) was used to catch up the specific bands, and the optical density of each band was measured using Image J software (National Institutes of Health, Bethesda, MD, USA).

### Transmission electron microscopy

The cells were harvested gently using trypsin-EDTA after treatment, followed by centrifugation, and the floating cells were collected. The cells were washed twice with cold PBS and fixed in 2% glutaraldehyde. After fixation, the cells were conventionally dehydrated, embedded, sectioned and stained, and the formation of autophagic vesicles was observed using TEM. During TEM study, 10 random fields were captured in a grid and the numbers of autophagic vesicles and cells were counted per field.

### Small interfering RNA transfection

The small interfering RNA (siRNA) was designed by GenePharma (GenePharma, Shanghai, China). The sequences of the Beclin-1 siRNA were as follows: sense, 5′- GGCACGAUCAAUAAUUUCATT-3′ antisense, 5′-UGAAAUUAUUGAUCGUGCCTT-3′. A negative control was also used: sense, 5′-UUCUCCGAACGUGUCACGUTT-3′ antisense, 5′-ACGUGACACGUUCGGAGAATT-3′. The siRNAs were transfected into cells with Lipofectamine 2000 (Invitrogen) according to the manufacturer's protocol. The transfection efficiency was confirmed by western blotting.

### Autophagy flux assay using mRFP-GFP-LC3

Cells were seeded in a 24-well plate and grown overnight. The cells were transfected with tandem mRFP/GFP-tagged LC3 using Lipofectamine 2000 according to the manufacturer's instructions. After a 24 h incubation, transfected cells were treated with the designated treatments. The cells were visualized with a fluorescence microscope. Because GFP is more sensitive to pH than RFP and is quenched at lysosomal pH, this construct can be used to quantify the rate of progression of autophagosomes (yellow puncta, GFP^+^/RFP^+^-LC3) to autolysosomes (red puncta, GFP^−^/RFP^+^-LC3) as a measure of the autophagic flux.^21, 49^ Autophagic vesicles were quantified by calculating the number of LC3 puncta (GFP^+^/RFP^+^-LC3 and GFP^−^/RFP^+^-LC3) in the images captured on the microscope.

### Immunofluorescence

Cells were fixed with 4% paraformaldehyde for 15 min at room temperature. After washing with PBS, the cells were permeabilized with 0.2% Triton X-100 for 5 min. After washing with PBS, sections were incubated in a blocking buffer containing 5% BSA for 30 min at room temperature, followed by incubations with anti-LC3 (1:200) and anti-p-ERK (1:500) antibodies overnight at 4 °C. Fluorescein-labeled secondary antibodies (Alexa Fluor488, Alexa Fluor647, 1:1000; Abcam) were applied for 2 h after which the cells were incubated with 0.1% DAPI for 5 min and washed with PBS. Microphotographs of LC3 and p-ERK fluorescence were captured on a wide-field fluorescent microscope. The detection of punctate LC3 staining from the diffuse staining indicated the formation of autophagosomes.

### Transient transfection and mitotracker red labeling

The cells were seeded in a 24-well plate, grown overnight and then transiently transfected with the GFP-LC3 plasmid using Lipofectamine 2000 according to the manufacturer's protocol to detect autophagy in mesangial cells,. The cells were then incubated for 24 h before treatment with the indicated drugs. After treatment, the cells were observed with a fluorescent microscope. The GFP-LC3 dots were considered autophagosomes. MitoTracker Red dye (Invitrogen) was added to the cell culture and incubated at 37 °C for 30 min to detect mitophagy in AGE-treated cells. Then the cells were observed with a fluorescent microscope. The colocalization of GFP-LC3 with MitoTracker Red was quantified as an index of mitophagy.

### Mitochondria isolation

Mitochondrial and cytosolic fractions were isolated from cells using a commercially available kit (Thermo Fisher Scientific, Waltham, MA, USA), according to the manufacturer's instructions. After fractionation, the pattern of Parkin expression was analyzed by western blotting.

### Statistical analysis

All experiments were repeated at least three times independently. The quantitative data are presented as the mean±S.E.M. Statistical analysis was performed using the Statistical Package for Social Science (SPSS) 17.0 software (SPSS, Chicago, IL, USA). Data in Figures 1, 2, 3, Figures 4c–f and Figure 5, 6, 7 were made using one-way analysis of variance, and Levene test was used for equality of variances, then the SNK test was used for comparisons between the two groups. Data in Figures 4a and b were performed using the *t*-test. All error bars show 95% confidence intervals: *P<*0.05 indicated that the observed difference was significant.

## Abbreviations

AGEs: advanced glycation end products
ROS: reactive oxygen species
Bcl-2: B-cell lymphoma-2
PARP: poly (ADP-ribose) polymerase
LC3: microtubule-associated protein 1 light chain 3
BSA: bovine serum albumin
MTT: 3-(4,5-dimethylthiazol-2-yl)-2,5-diphenyl tetrazolium bromide
PI: propidium iodide
JC-1: 5,5′,6, 6′ tetrachloro-1,1′,3,3′-tetraethylbenzimidazolylcarbocyanine iodide
CCCP: carbonyl cyanide 3-chlorophenylhydrazone.
