# Supplementary figures and images for: Inhibition of autophagy increased AGE/ROS-mediated apoptosis in mesangial cells
Source: Cell Death Dis. 2016 Nov 3;7(11):e2445–. doi: 10.1038/cddis.2016.322 (PMC5260901; doi:10.1038/cddis.2016.322)

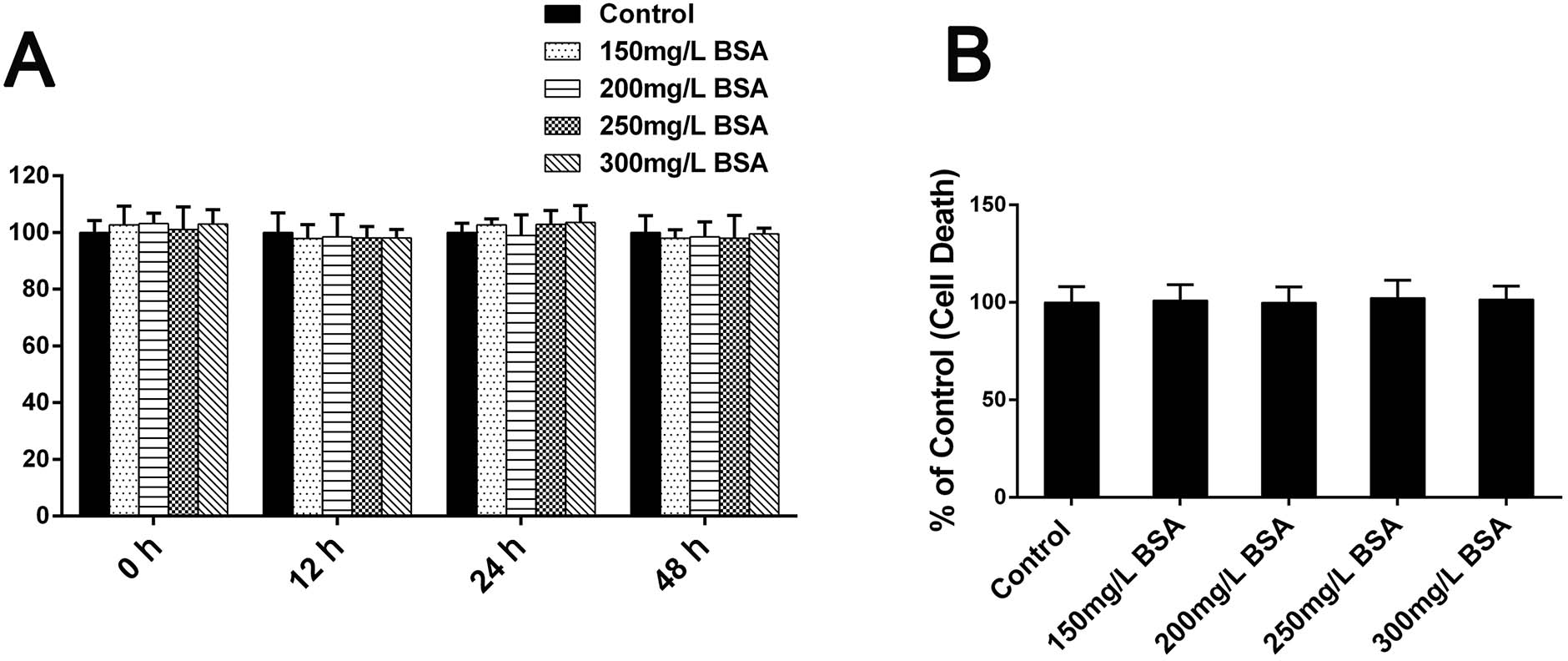

Supplement: Supplementary Figure 1 [file cddis2016322x1.tif]
