# Supplementary material for: Inhibition of autophagy increased AGE/ROS-mediated apoptosis in mesangial cells
Source: Cell Death Dis. 2016 Nov 3;7(11):e2445–. doi: 10.1038/cddis.2016.322 (PMC5260901; doi:10.1038/cddis.2016.322)
Supplement: Supplementary Information [file cddis2016322x2.doc]

**SUPPLEMENTAL FIGURE LEGENDS**

**Supplemental Figure S1. The effect of BSA on mesangial cells.** (A). Cells were treated with BSA at various concentrations (150-300mg/L) for 0, 12, 24, or 48 ~~H~~ h, and cell viability was estimated using MTT assay. Data are presented as mean ± S. E. M. of at least three independent experiments. (B). Cells were treated with BSA at various concentrations (150-300mg/L) for 24 ~~H~~ h. Cell death was estimated using a cell death detection ELISA^PLUS^ assay. Data are presented as mean ± S. E. M. of at least three independent experiments.
